# Intersectin (ITSN) Family of Scaffolds Function as Molecular Hubs in Protein Interaction Networks

**DOI:** 10.1371/journal.pone.0036023

**Published:** 2012-04-27

**Authors:** Katy A. Wong, Jessica Wilson, Angela Russo, Li Wang, Mustafa Nazir Okur, Xuerong Wang, Negin P. Martin, Erica Scappini, Graeme K. Carnegie, John P. O'Bryan

**Affiliations:** 1 Department of Pharmacology, University of Illinois College of Medicine, Chicago, Illinois, United States of America; 2 Center for Cardiovascular Research, University of Illinois College of Medicine, Chicago, Illinois, United States of America; 3 UIC Cancer Center, University of Illinois College of Medicine, Chicago, Illinois, United States of America; 4 Department of Biochemistry and Molecular Genetics, University of Illinois College of Medicine, Chicago, Illinois, United States of America; 5 Laboratory of Neurobiology, National Institute of Environmental Health Sciences, National Institutes of Health, Department of Health and Human Services, Research Triangle Park, North Carolina, United States of America; Purdue University, United States of America

## Abstract

Members of the intersectin (ITSN) family of scaffold proteins consist of multiple modular domains, each with distinct ligand preferences. Although ITSNs were initially implicated in the regulation of endocytosis, subsequent studies have revealed a more complex role for these scaffold proteins in regulation of additional biochemical pathways. In this study, we performed a high throughput yeast two-hybrid screen to identify additional pathways regulated by these scaffolds. Although several known ITSN binding partners were identified, we isolated more than 100 new targets for the two mammalian ITSN proteins, ITSN1 and ITSN2. We present the characterization of several of these new targets which implicate ITSNs in the regulation of the Rab and Arf GTPase pathways as well as regulation of the disrupted in schizophrenia 1 (DISC1) interactome. In addition, we demonstrate that ITSN proteins form homomeric and heteromeric complexes with each other revealing an added level of complexity in the function of these evolutionarily conserved scaffolds.

## Introduction

The regulation of biochemical pathways is mediated in part through numerous protein-protein interactions that are facilitated by protein scaffolds. It has become widely accepted that protein interactions are frequently mediated by modular protein recognition domains with characteristic binding and functional properties. For example, the Src homology 2 (SH2) domain represents the prototypical modular domain responsible for interaction with phosphotyrosine. Since the discovery of SH2 domains, a plethora of domains have been defined, each with unique specificity for distinct types of ligands (reviewed in [1]). The arrangement of these protein interaction domains results in intricate interaction networks for a given protein.

Intersectin (ITSN) is a multi-domain scaffold protein. There are two *ITSN* genes in mammals, *ITSN1* and *ITSN2*, each encoding a short and long isoform and sharing 59% identity and greater than 70% similarity. Both ITSN short (ITSN-S) isoforms possess two amino-terminal Eps15 homology (EH) domains followed by a coiled-coil (CC) domain and five Src homology 3 (SH3) domains. The ITSN long isoform (ITSN-L) contains all these domains in addition to an extended carboxy-terminus encoding a Dbl homology (DH) domain, a Pleckstrin homology (PH) domain, and a C2 domain. The DH and PH domains function together as a guanine nucleotide exchange factor (GEF) that regulates the activation of the Rho family GTPase Cdc42. In addition to these major isoforms, there are numerous additional splice variants for each ITSN, several of which have altered interactions with specific targets [2], [3].

The modular domains present in ITSN initially suggested a role in endocytosis and indeed, ITSNs interact with numerous components of the endocytic machinery to regulate the processes of endocytosis [2]. However, ITSNs also associate with a number of proteins involved in regulating signal transduction pathways suggesting additional functions for these scaffold proteins [2]. Although a number of ITSN binding proteins have been described, we sought to identify novel ITSN targets through use of a high throughput yeast two-hybrid screen. The advantage of our approach is that we used smaller fragments of ITSN (<350aa) encompassing one or two individual domains of the protein thereby allowing for identification of interacting proteins specific to each of these modular domains. We have identified an extensive array of binding proteins for both ITSN1 and ITSN2 and present an analysis of several novel ITSN targets. In addition to identification of known pathways regulated by ITSNs, our findings reveal a number of novel biochemical pathways involving ITSNs.

## Materials and Methods

### Cell lines and reagents

COS cells (kindly provided by Dr. John Cidlowski, NIH) [4] and HEK 293T cells (kindly provided by Dr. Brian Howell, Fred Hutchinson Cancer Center) [5] were maintained in Dulbecco's Modified Eagle Medium (DMEM) supplemented with 10% fetal bovine serum. COS cells were transfected with Lipofectamine (Invitrogen, Carlsbad, CA) as recommended by the manufacturer. HEK293T cells were transfected with the calcium phosphate procedure as described previously [6]. Antibodies used include anti-hemagglutinin (HA) monoclonal (Covance, Emeryville, CA), anti-FLAG monoclonal (Sigma, St. Louis, Missouri), anti-Rab5 monoclonal (Santa Cruz Biotechnology, Santa Cruz, CA), anti-VSV (Sigma, St. Louis, Missouri), anti-EEA1 goat polyclonal (Santa Cruz Biotechnology, Santa Cruz, CA), anti-ITSN1 rabbit polyclonal [4], anti-MYC monoclonal (Sigma, St. Louis, Missouri). Glutathione-Sepharose beads were purchased from GE Healthcare (Piscataway, NJ)

**Figure 1:**
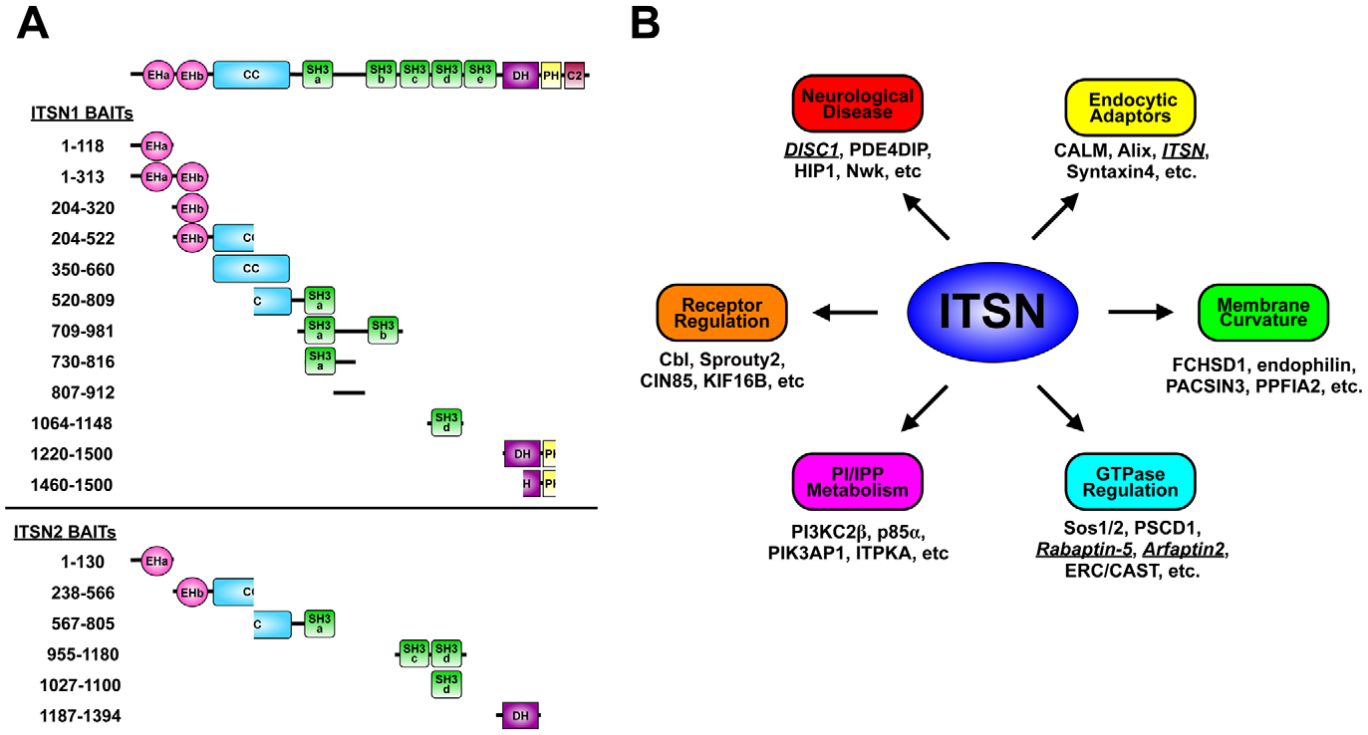
Yeast two-hybrid (Y2H) analysis of ITSN scaffolds. A. ITSN baits. Shown are schematic representations of the long isoforms of ITSN1 and ITSN2 with the regions used in Y2H screening shown under each structure. Baits encompassing the DH-PH-C2 regions of ITSN2 were not successful in screening. B. Targets of ITSN proteins identified by Y2H and the biological processes regulated by these targets. Those targets characterized in this manuscript are underlined and italicized.

### DNA Constructs

The BiFC vectors pFlag-VN-173N and pHA-VC155N were gifts from Dr. Chang-Deng Hu (Purdue University, West Lafayette, IN). The Rab5 constructs were gifts from Dr. Richard Pagano (Mayo Clinic, Rochester, MN). DISC1 constructs were gifts from Dr. Jill Morris (Northwestern University, Chicago, IL). The human ITSN2 constructs were gifts from Susana de la Luna (Center for Genomic Regulation, Barcelona, Spain). The Arf6 and Arfaptin2 constructs were gifts from Dr. John Exton (Vanderbilt University, TN). The PDE4D3 construct was a gift from Drs. George Baillie and Kimberly Dodge-Kafka (University of Glasgow, Glasgow, UK).

### Yeast two-hybrid screens

Yeast two-hybrid (Y2H) analysis was performed through a contract with Myriad Genetics essentially as described previously [7] using the various individual domains of mouse or human ITSN as bait. The various ITSN baits were used to screen multiple cDNA libraries derived from human or mouse tissues as indicated in Tables S1 and S2.

### Bimolecular fluorescence complementation (BiFC) and confocal imaging

BiFC was performed essentially as described [8]. Briefly, expression constructs consisting of the amino-terminus or carboxy-terminus of Venus (VN and VC, respectively) [9] were fused to the amino-terminus of the respective proteins interest. COS cells were transiently transfected late in the day and then imaged the following morning so that the various proteins were not vastly overexpressed. CFP was co-transfected with the BiFC constructs, at a fivefold-lower concentration, to mark transfected cells. Samples were then imaged by confocal analysis on a Zeiss LSM 510 confocal microscope as described [4], [8]. Note that cells transfected with either VN– or VC– fusions alone were not fluorescent due to lack of the corresponding complementary fragment [8]. As a negative control, VN-fusion constructs were co-transfected with VC-pep which expresses an 11 amino acid peptide (TSRLPPLGVGN) fused to the carboxy-terminus of VC.

### Immunofluorescent staining of cells

Cells were plated on glass bottom plates, fixed in 3.7% formaldehyde, then permeabilized by incubation in blocking buffer (1xPBS, 3% BSA, 0.1% Triton X-100) for 1 hr at RT. Primary antibody was added for 2 hrs at room temperature, rinsed 3 times with 1xPBS. Fluorescently tagged secondary antibody (donkey anti-rabbit, anti-mouse, or anti-goat antibodies conjugated to either fluorescein isothiocyanate (FITC), tetramethylrhodamine B isothiocyanate (TRITC), or Cy5; Jackson ImmunoResearch, West Grove, PA) was added to samples for 1 hr at room temperature followed by 3 rinses with 1xPBS. Samples were imaged on a confocal microscope as described above.

### GST pull down experiments

GST-ITSN1 fusion proteins were used to affinity purify specific targets from cell lysates as previously described [10]. Briefly, GST-ITSN1 encompasses the entire ITSN1-S coding region, GST-EH encompasses both EH domains, GST-CC encompasses the coiled-coil (CC) region, GST-EH-CC encompasses both EH domains and the CC region, and GST-SH3 encompasses all five SH3 domains. HEK293T cells were transiently transfected with the indicated expression constructs. Forty-eight hours after transfection, cells were washed with warm phosphate-buffered saline (PBS) and lysed in PLC lysis buffer (50 mM HEPES, pH 7.5, 150 mM NaCl, 10% glycerol, 1% Triton X-100, 1 mM EGTA, 1.5 mM MgCl2, and 100 mM NaF) supplemented with protease inhibitors. After 30 min at 4°C on a nutator, the soluble lysates were obtained by centrifugation at 14,000 rpm for 10 min and used for the GST pull-down assay. GST fusion proteins were purified from DH5α E. coli using glutathione-sepharose beads. Equivalent amounts of fusion proteins along with GST as a negative control were added to equal amounts of cell lysates, incubated on a nutator at 4°C for 2 h. At the end of the incubation, beads were spun down, washed three times with PLC lysis buffer (described above) and then resuspended in LDS sample buffer (Invitrogen, Carlsbad, CA). The samples were fractionated on a gel, transferred to an Immobilon-P membrane, and probed with the indicated antibodies. The blots were developed with SuperSignal chemiluminescence reagent (Pierce, Rockford, IL).

## Results

### Identification of ITSN targets by high throughput yeast two-hybrid (Y2H) analysis

In an effort to define the biological targets of the ITSN scaffolds, we subjected ITSN1 and ITSN2 to a high throughput Y2H screen. To identify ligands of each ITSN domain, our approach utilized ITSN bait fragments encoding subsets of the domains present in ITSN and spanning the majority of the coding sequence of both proteins (Figure 1A). Furthermore, we screened multiple prey libraries generated from human brain, spleen, macrophage, and skeletal muscle as well as mouse embryo. The ITSN binding proteins identified in our screen are listed in Tables S1 and S2 with targets unique to this study highlighted in yellow. We identified 55 binding proteins for ITSN1, 62 interactors for ITSN2, and 10 targets common to both ITSNs. However, our screen failed to isolate several validated targets of ITSNs suggesting that our results underestimate the number of *bona fide* ligands *in vivo*. For example, ITSN1 binds the E3 ubiquitin ligase Cbl [4], which was identified as a target in the ITSN2 screen but not in the ITSN1 screen (see Tables S1 and S2). The pool of ITSN binding proteins highlights the pivotal role of ITSNs in endocytosis, GTPase regulation, and receptor tyrosine kinase (RTK) regulation (Figure 1B), and suggests that ITSNs may have novel roles in regulating additional pathways as detailed in the following sections.

**Figure 2:**
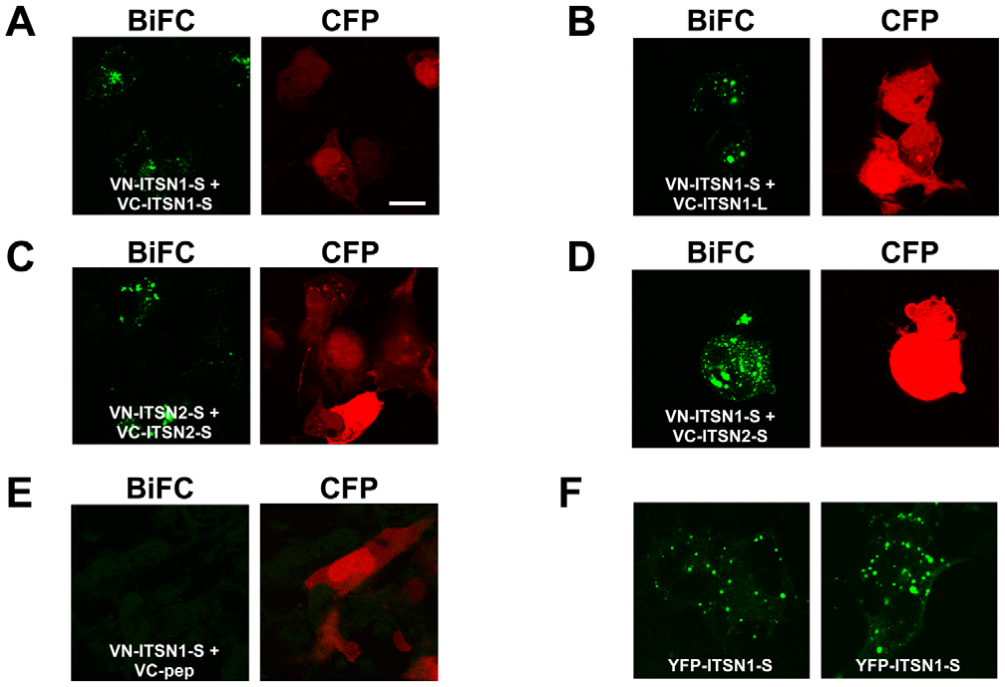
ITSNs form homo and heterodimers. Bimolecular fluorescence complementation was used to assess the dimerization of ITSN1 and ITSN2 isoforms in COS cells. A–E. The BiFC emission from the reconstituted Venus fluorophore was pseudocolored green (left panel). CFP (pseudocolored red, right panel) was co-transfected to mark transfected cells and only CFP-positive cells were imaged BiFC. A. ITSN1-S forms homodimers. VN-ITSN1-S was co-expressed with VC-ITSN1-S. B. The short and long isoforms of ITSN1 form heterodimers. VN-ITSN1-S was co-expressed with VC-ITSN1-L. C. ITSN2-S forms homodimers. VN-ITSN2-S was co-expressed with VC-ITSN2-S. D. ITSN1 and ITSN2 heterodimerize. VN-ITSN1-S was co-expressed with VN-ITSN2-S. E. As a negative control for BiFC, VN-ITSN1-S was co-expressed with a non-specific peptide fused to VC (VC-pep). F. For comparison with the BiFC results, YFP-ITSN1-S was expressed in COS cells. Shown are two different panels of positive cells. The fluorescence patterns shown in all panels (A–F) are representative of localization observed throughout the plate. These results are representative of at least three independent experiments. Note: white scale bar represent 10? m.

### ITSN-targets involved in endocytosis

Twenty-one proteins involved in endocytosis were identified as potential ITSN binding proteins in our Y2H screens (Tables S1 and S2) consistent with ITSNs' known role in this process [2], [11], [12]. Several of these targets have previously been identified as ITSN partners including dynamin II, Epsin2, Eps15, Eps15L, clathrin, synaptojanin2b, amphiphysin, SNAP29, HIP1, Dab2, PACSIN3, AP2, REPs, CIN85, and SH3GL1 (EEN) (reviewed in [2], [11]). However, a number of additional endocytic proteins were identified as potential ITSN1 targets in our screen including CALM, KIF16B, Alix, FNBP1, and Syntaxin4. These results further support the involvement of ITSNs in regulating endocytosis [2].

### Dimerization of ITSNs

ITSN1 was isolated as a binding partner for itself (Tables S1) suggesting that ITSN1, and potentially ITSN2, may form homo– and heterodimers *in vivo*. Given that related EH-domain containing protein Eps15 and its family member Eps15L form tetramers [13], [14], we tested whether ITSNs also formed oligomeric complexes. Since initial attempts to co-precipitate ITSNs tagged with different epitope tags (e.g., GFP and HA) were not successful, we turned to using bimolecular fluorescence complementation (BiFC) [15] to assess interactions. The advantages of this technique are that it allows for visualization of protein-protein interactions in live cells and does not require high overexpression of proteins or elaborate post-processing of images as seen with other techniques such as fluorescence resonant energy transfer (FRET). ITSN1-S was fused to the carboxy-terminus of amino acids 1–173 of Venus (VN) or amino acids 155–238 of Venus (VC) [16]. Both VN-ITSN1-S and VC-ITSN1-S were co-expressed in COS cells resulting in fluorescence complementation demonstrating for the first time that ITSN1 forms oligomers (Figure 2A). The intracellular distribution of the BiFC signal was indistinguishable from that of YFP-ITSN1-S (Figure 2F) [10], [17]. Given that ITSN1-L contains all the domains of ITSN1-S, we also examined the ability of ITSN1-L to homodimerize and heterodimerize with ITSN1-S. ITSN1-L formed homodimers (data not shown) and also formed heterodimers with ITSN1-S (Figure 2B). Given the conserved structures of ITSN1 and ITSN2 and the high degree of sequence homology between these two proteins, we tested whether ITSN2 homodimerized as well as heterodimerized with ITSN1. Like ITSN1, ITSN2-S formed homomeric complexes (Figure 2C) as well as heteromeric complexes with ITSN1-S (Figure 2D). No signal was observed upon co-expression of VN-ITSN1-S with VC-pep, a non-specific peptide fused to VC as a negative control (Figure 2E). These data indicate that ITSN1 and ITSN2 isoforms form a variety of homo- and heterodimeric complexes.

**Figure 3:**
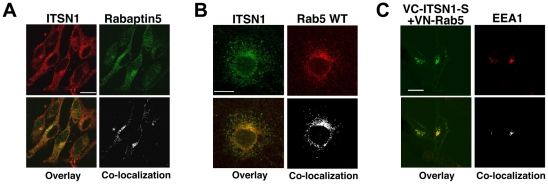
ITSN1 interacts with components of the Rab5 GTPase pathway. A. ITSN1 interacts with Rabaptin5. Endogenous ITSN1 (red) in HeLa cells co-localizes with myc-tagged Rabaptin5 (green). Antibodies to Rabaptin5 did not detect the endogenous protein. Overlap of proteins is shown in yellow. Regions of overlap were extracted from the image using ImageJ. B. Endogenous ITSN1 (green) co-localizes with endogenous Rab5 (red) in COS cells. Overlap of proteins is shown in yellow. Regions of overlap were extracted from the image using ImageJ. C. ITSN1-S forms a complex with Rab5 on early endosomes. HeLa cells were transfected with VN-Rab5 and VC-ITSN1-S and then stained with antibody to EEA-1 (red). The BiFC complex of Rab5 and ITSN1-S is pseudocolored green. Overlap of the BiFC complex with EEA1 is shown in yellow. Regions of overlap were extracted from the image using ImageJ. Note: white scale bars represent 20? m. The fluorescence patterns shown in all panels are representative of localization observed throughout the plate.

### GTPase regulation

Previous studies demonstrated that ITSN1 regulates activation of several members of the Ras superfamily including Ras [17], [18], Cdc42 [18], [19], [20], and potentially Rac [21]. Our Y2H screen identified a number of regulators and effectors of multiple Ras family GTPases including Ras, Rab, Arf, and Rho (Tables S1 and S2). ITSN1 has previously been reported to bind Sos1, a Ras GEF, leading to Ras activation [17], [22], [23]. In addition to reproducing these findings, we isolated both Sos1 and Sos2 as ITSN2 binding partners suggesting that both ITSN1 and ITSN2 participate in Ras activation via their interaction with multiple Ras GEFs. Exchange factors for additional Ras family members were also isolated in our Y2H screen including TRIO (Rho GEF), P-REX1 (Rac GEF), TIAM1 (Rac GEF), SWAP70 (Rac GEF), and PSCD1/cytohesin (Arf GEF). In addition to GEFs, ITSNs interacted with several GTPase activating proteins (GAPs) including the p85α subunit of PI3Ks (Rab GAP) [24] and HRB/HRB-L (Arf GAPs). Although not a GAP, PPFIA2 binds GIT1, a G-protein coupled receptor (GPCR)-interacting ArfGAP [25]. Finally, ITSNs also interacted with a number of effectors and targets of Ras superfamily GTPases including Arfaptin2 (Arf and Rac), SCOCO (Arfs), GOLGA4 (Rabs), GolgB1 (Rabs), Rabaptin-5 (Rabs), KIF16B (Rabs), and ERC1/CAST (Rabs). Both Arf and Rab play integral roles in regulating vesicular trafficking providing a potential mechanistic link with ITSNs' established role in endocytosis and receptor trafficking [4]. Thus, ITSNs' ability to interact with multiple regulators of the Arf and Rab pathways suggest an *in vivo* link of ITSNs to regulation of these Ras family GTPase.

To confirm a subset of these interactions, we examined the interaction of ITSN1 with components of the Rab and Arf pathways (Figure 3). Endogenous ITSN1 co-localized with the Rab-5 regulator, Rabaptin-5 (Figure 3A). Initial attempts to stain for endogenous Rabaptin-5 were unsuccessful due to background problems with available antibodies [26]. Given the involvement of Rabaptin-5 in the regulation of Rab5 activation and function [26], we examined whether ITSN1 interacted with Rab5. Endogenous ITSN1 co-localized with endogenous Rab5 (Figure 3B). BiFC analysis confirmed an interaction between these two proteins (Figure 3C). Furthermore, the ITSN1-Rab5 complex localized with early endosomal antigen 1 (EEA1) indicating that the ITSN1-Rab5 complex was associated with early endosomes (Figure 3C).

**Figure 4:**
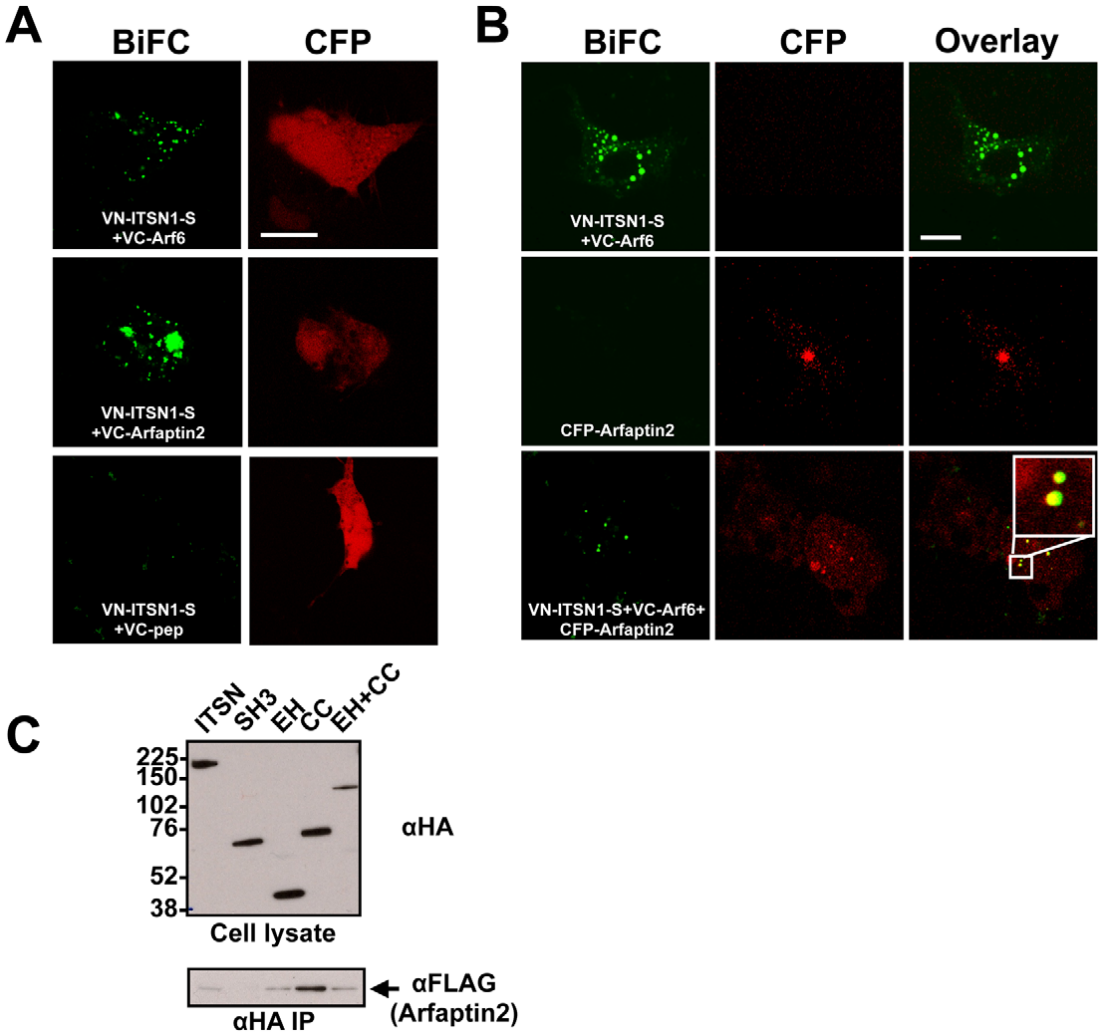
ITSN1 interacts with components of the Arf6 GTPase pathways. A. ITSN1 interacts with Arf6 as well as its effector Arfaptin2. BiFC was used to analyze the interaction of ITSN1S with components of the Arf6 pathway. VN-ITSN1-S was co-expressed in COS cells with VC-Arf6 (top panels), VC-Arfaptin2 (middle panels), or VC-pep (bottom panels) as a negative control. The emission from the reconstituted Venus fluorophore was pseudocolored green. CFP (pseudocolored red) was co-transfected to mark transfected cells and only CFP-positive cells were imaged. Scale bar, 20 um. The fluorescence pattern shown in all panels are representative of localization observed throughout the plate. B. ITSN1 forms a trimolecular complex with Arf6 and Arfaptin2 in COS cells. Top panels: VN-ITSN1-S and VC-Arf6 were co-expressed in the absence of CFP-Arfaptin2 to demonstrate lack of bleed through of the BiFC signal into the CFP channel. Middle panels: CFP-Arfaptin2 was expressed alone to demonstrate lack of bleed through of the CFP signal into the BiFC channel. Bottom panels: VN-ITSN1-S, VC-Arf6, and CFP-Arfaptin2 were co-expressed in COS cells. The BiFC complex of VN-ITSN1-S and VC-Arf6 co-localizes with CFP-Arfaptin2. A region of overlap of the BiFC complex with CFP-Arf6 is enlarged in the inset in the bottom right panel. Note: white scale bar represents 20? m. The fluorescence pattern shown in all panels are representative of localization observed throughout the plate. C. VC-Arfaptin2 co-precipitates with ITSN1. HA-epitope tagged ITSN1-S or various domains of ITSN1 were co-expressed with VN-Arfaptin2 which contains a FLAG epitope tag. HA-immunoprecipitates were probed for either HA (top panel) or FLAG (bottom) to detect association of Arfaptin2. The CC region interacts most strongly with VN-Arfaptin2 consistent with the Y2H results (see Tables S1).

In addition to identification of Rab pathway components, our Y2H screen identified potential links between ITSN1 and multiple components of the Arf pathway as noted above. To explore the potential interaction of ITSN1 with the Arf pathway, we utilized BiFC to examine interaction of ITSN1 with Arfaptin2, an effector of Arf6 and Rac [27], [28]. As shown in Figure 4A, ITSN1 associates with Arf6 and its effector Arfaptin2. In addition, ITSN1, Arf6, and Arfaptin2 co-localize on a subset of structures (Figure 4B) which we presume represent intracellular vesicles given their punctate appearance and similar morphology to EGFR and ITSN1 double positive vesicles [17] as well ITSN1-Rab5 positive structures (Figure 3). Finally, co-expression of Arfaptin2 with the full length ITSN1 or various truncation mutants indicates that Arfaptin2 interacts with ITSN1 through the EH domains and CC region (Figure 4C). Although Arfaptin2 appears to bind more weakly to the EH-CC fragment, this protein is expressed at lower levels. Thus, it appears that Arfaptin2 interacts similarly with the CC and EH-CC fragments suggesting that the CC region is the predominant region for interaction consistent with our Y2H results (see Tables S1). These data indicate that ITSN1 is a component of Arf GTPase regulated pathways.

### ITSNs and receptor tyrosine kinase regulation

While the above interactions demonstrate a role for ITSNs in GTPase regulated pathways in particular, our Y2H results reveal additional links between ITSNs and regulators of signal transduction pathways (Tables S1 and S2). For example, ITSNs interacted with both Cbl and phosphatidylinositol 3-kinase, specifically PI3K-C2β (Tables S1 and S2). Indeed, ITSN1 regulates both of these proteins [4], [10]. However, our Y2H screens identified additional components of RTK pathways as potential binding partners for ITSNs. More specifically, we identified several regulators of the E3 ubiquitin ligase Cbl: Alix, Sprouty2 (Spry2) and CIN85. Interestingly, CIN85 has been described as an ITSN1 target [29] and we have recently demonstrated that Spry2 represents a *bona fide* target of ITSN1 [30].

**Figure 5:**
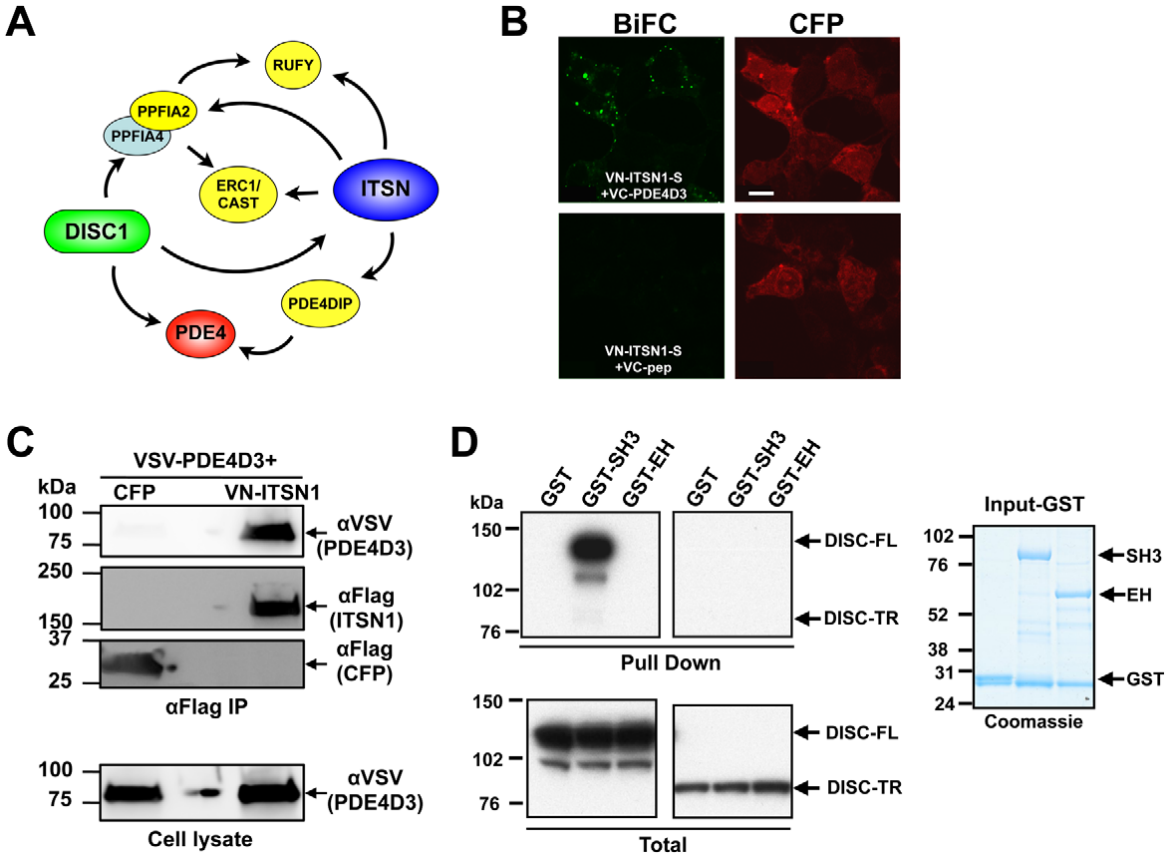
ITSNs and DISC1 interact with common proteins. A. ITSN1 was identified as a binding partner for DISC1 by Y2H [31]. However, a number of additional connections between ITSN1/2 and DISC1 were identified in our Y2H screen. Proteins shaded yellow were identified as ITSN1 or 2 binding partners by Y2H. PPFIA2 is a member of the liprinα family of scaffolds (PPFIA1-4) which bind the LAR family of transmembrane protein phosphatases and are known to form heteromeric complexes with each other [57]. Thus, ITSNs and DISC1 may interact through a heteromeric complex of PPFIA2 and PPFIA4. PDE4 (red), a known DISC1 interacting protein, was examined for interaction with ITSN1. B. Interaction of ITSN1 with phosphodiesterase 4D3 isoform. HEK293T cells grown on coverslips were transfected with VN-ITSN1-S and either VC-PDE4D3 or VC-pep as a negative control. CFP was co-transfected to mark transfected cells and only CFP-positive cells were imaged. The fluorescence pattern shown is representative of localization observed throughout the plate. Note: white scale bar represent 10? m. C. ITSN1-S and PDE4D3 interaction was confirmed by co-immunoprecipitation. HEK293T cells were transfected with VSV-tagged PDE4D3 and either VN-ITSN1-S or CFP. ITSN1 and CFP were immunoprecipitated with FLAG antibody and the specific co-immunoprecipitation of PDE4D3 was determined by Western blot analysis with αVSV antibody. Input (bottom panel labeled “Cell lysate") shows the level of PDE4D3 was expressed equally in both cell lysates. Note that an empty lane was between the CFP and VN-ITSN1-S samples on the gel. The weak signal for PDE4D3 in that lane resulted from overflow of the sample. The top 3 panels in C were from the same membrane which had been separated into the indicated size ranges and probed with the indicated antibodies. CFP and VN-ITSN1-S each possess a FLAG epitope tag. Similar results were obtained from three independent experiments. D. ITSN1 SH3 domains bind DISC1. HEK293T cells were transfected with V5-tagged full length DISC1 (DISC1 FL; aa 1–854) or a truncated DISC1 (DISC1-TR; aa 1–597) corresponding to the deletion resulting from a translocation breakpoint that disrupts the DISC1 locus [58]. GST-SH3 (encoding all 5 SH3 domains) but not GST or GST-EH (encoding both EH domains) pulls down DISC1-FL but not DISC 1-TR (top panels). Expression of DISC1 proteins in cell lysates is shown in the Western blot of cell lysates with αV5 antibody (bottom panels). Input GST fusion proteins are indicated in the Coomassie-stained gel to the far right.

### ITSNs and neurological disease

ITSN1 has been implicated in neurodegeneration through its link with regulation of the JNK MAPK pathway and its association with several proteins that interact with the protein product of the Huntington's Disease gene (reviewed in [2]). Furthermore, ITSN1 was identified as a binding partner for DISC1 (disrupted in schizophrenia 1) [31] suggesting a potential role in psychiatric disease as well [31]. Indeed, several of the identified ITSN1 and ITSN2 targets interact with a DISC1 binding protein (e.g., PDE4DIP, ERC1, PPFIA2, and RUFY) (Figure 5A). Given DISC1's interaction with phosphodiesterase 4 [32], we tested whether ITSN1 might also interact with this pathway. Co-expression of VN-ITSN1-S with VC-PDE4D3 resulted in significant BiFC signal whereas the VC-pep control was negative (Figure 5B). To confirm this interaction, we further examined the association of ITSN1 and PDE4D3 by co-immunoprecipitation. PDE4D3 co-precipitates with FLAG-tagged ITSN1-S (VN-ITSN1-S) but not with FLAG-tagged CFP (Figure 5C). Given this association of ITSN1 with PDE4D3, we next tested whether ITSN1 might directly interact with DISC1. Examination of the DISC1 sequence indicates the presence of a Pro-rich sequence (aa 730–736; PPIPPRL) that conforms to a consensus ITSN1 SH3 binding site [10]. Using GST-tagged fusion proteins, we observed significant binding of ITSN1's SH3 domains to DISC1 (Figure 5D). However, neither GST nor GST-EH interacted with DISC1 demonstrating specificity in this interaction. Furthermore, we did not detect significant binding of ITSN1 SH3 domains to the truncated DISC1 protein which lacks the consensus Pro-rich sequence. Together, these data suggest that ITSN1 and possibly ITSN2, may coordinate interaction of several proteins with the DISC1.

### Additional functions

#### Membrane curvature

ITSNs bind FCHo proteins at sites of nascent vesicle formation [33] suggesting a role for ITSNs in regulating membrane curvature [2]. In further support of this notion, we identified multiple ITSN binding partners involved in membrane curvature including Arfaptin2 (Figure 4), FCHSD1 & 2, CALM, Epsin2a, HIP1, PACSIN3, FNBP1, endophilin, amphiphysin, FNBP4, and PPFIA2 suggesting that ITSNs may induce or maintain membrane curvature through interactions with this class of proteins.

#### Inositol phosphate/phosphatidylinositol phosphate metabolism

ITSN1 binds a novel class 2 phosphatidylinositol 3-kinase, PI3K-C2β, to regulate an AKT cell survival pathway in neurons [10]. Additionally, our Y2H results reveal connections of ITSNs with multiple proteins involved in regulating inositol/phosphatidylinositol metabolism (Table 1). In addition to PI3K-C2β, ITSNs bind the p85α regulatory subunit of class 1 PI3Ks, Beclin-1, a target for Class 3 PI3K, and PIK3AP1, an adaptor protein that links the B-cell receptor to the p85 subunit of Class 1 PI3Ks. These interactions suggest an involvement of ITSNs in regulation of multiple classes of PI3Ks. The isolation of inositol 1,4,5 trisphosphate 3-kinase A (ITPKA) as an ITSN1 binding protein suggests a role for ITSNs in production of inositol phosphates as well. In addition to regulating kinases that produce inositol or phosphatidylinositol products, ITSNs may also regulate the dephosphorylation of these important signaling molecules. ITSN1 interacts with two inositol 5-phosphatases, SHIP2 [34], [35] and synpaptojanin [36], [37], the latter of which was identified in our Y2H screen (Tables S1) and plays an important role in membrane curvature and membrane dynamics through regulation of PI(4,5)P_2_ levels [38]. Our results reveal an additional interaction with the phosphatidylinositol 3-phosphatase, myotubularin through the intermediate filament protein desmin (Table 1) suggesting that ITSNs may regulate a dynamic network of proteins controlling the flux of inositol phosphates at different stages of endocytosis. Indeed, the ITSN1-SHIP2 interaction is important for regulation of endosomal dynamics [34].

## Discussion

Previous studies have utilized Y2H screens to identify ITSN binding partners [39], [40]. Although the number of isolated ITSN targets in Stelzl *et. al*. was quite limited (six ITSN1 targets; three ITSN2 targets)[39], Wang and colleagues [40] identified 60 putative targets of *C. elegans* ITSN1 in their Y2H screen, a number of which overlap with our results (e.g., CIN85/Ruk, Alix, epsin, Dab, dynamin and synaptojanin). In contrast to these previous screens, we employed a high throughput approach using smaller fragments of ITSNs (<350aa) encompassing one or two of the individual domains of ITSNs. This approach allowed for the identification of interacting proteins specific to each of the modular domains present in ITSNs. Indeed, our screens identified a total of 127 potential ITSN-binding proteins, with the majority of these putative ITSN-binding proteins representing novel targets for the ITSN scaffolds. Although we believe that our results provide a more comprehensive list of targets for the ITSN proteins, it is possible that some of these proteins may not interact with full length ITSNs. Thus, additional work will be needed to validate each of these new targets.

ITSNs are evolutionarily conserved scaffold proteins that were initially implicated in regulating endocytosis based on their domain homology with other EH-containing proteins involved in this process [36], [41], [42]. Indeed, ITSNs interact with multiple endocytic proteins [37], [41], [43], [44] and alter endocytosis when overexpressed in cells [41], [45]. Furthermore, loss-of-function mutations in ITSN1 lead to clear defects in endocytosis [40], [46], [47], [48], [49]. These defects appear to be due to impaired recruitment of endocytic proteins to sites of membrane curvature induced by FCHo proteins [33]. Our Y2H experiments reveal that ITSNs interact with numerous proteins involved in membrane curvature including Arfaptin2 (Figure 4), FCHSD1 & 2, CALM, Epsin2a, HIP1, PACSIN3, FNBP1, endophilin, amphiphysin, FNBP4, and PPFIA2. Thus, ITSNs may play a critical role in the induction or maintenance of membrane curvature through interaction with this class of proteins.

**Table 1:** Regulators of inositol/phosphatidylinositol metabolism.

Target	Function
PI3K-C2β	Class 2 PI3K
p85a	adaptor for Class 1 PI3K
Beclin 1	regulatory subunit of Class 3 PI3K
PIK3AP1	binds p85 subunit of Class 1 PI3Ks
ITPKA	Ins(1,4,5)P_3_ 3-kinase
Desmin	intermediate filament protein; binds and regulated by MTM1 3-lipid phosphatase
SYNJ	inositol 5-phosphatase and polyphoshoinositol phosphatase
SHIP2	inositol 5-phosphatase

While ITSNs clearly play a role in the process of endocytosis, accumulating studies demonstrate additional biochemical roles for these scaffolds (reviewed in [2]). Consistent with these studies, our Y2H results reveal potential new connections between ITSNs and several cellular pathways including Rab and Arf GTPase regulation, receptor tyrosine kinase regulation, and inositol phosphate/phosphatidylinositol metabolism (Table 1). Although these targets represent distinct biochemical pathways, there is also clear overlap of these pathways with the endocytic pathway. Indeed, endocytosis plays a critical role in regulation of many RTKs [50]. Several of the ITSN-binding proteins we identified (Alix, Spry2, CIN85) represent attractive ITSN targets that might provide an explanation for our previous results that demonstrate ITSN1 enhances Cbl activity toward ubiquitylating the EGFR [4]. Although the precise mechanism by which ITSN1 enhances Cbl function remains ill-defined, ITSN1 might enhance the interaction of Cbl with an activator or disrupt interaction of Cbl with an inhibitor [4]. Indeed, Spry2 represents a *bona fide* target of ITSN1 that inhibits activation of Cbl. These results suggest that ITSN1 enhances Cbl activation by disrupting the interaction of Spry2 with Cbl [30].

Regulation of inositol phosphate/phosphatidylinositol metabolism also has important implications for the endocytic pathway as well [51]. In addition, ITSNs may also play a role in regulating nuclear processes through the interaction with a diverse array of nuclear proteins including RNA binding proteins and transcription factors (Tables S1 and S2). Together, these results suggest that ITSN scaffolds function as critical biochemical hubs within the cell [52]. However, it is most likely that ITSNs function to fine-tune various biochemical pathways rather than function as obligate components. Indeed, loss-of-function mutations in ITSN in both mouse and *C. elegans* are viable and result in very mild mutant phenotypes with subtle defects in endocytosis [40], [48], [49].

Many of the published binding partners for ITSNs interact with the same regions raising the question of how ITSN's specifically interact with each of these targets. For example, Cbl, Sos, PI3K-C2β, WASP, and Numb all interact specifically, although not exclusively, with the SH3A domain of ITSN1 [2]. Although, it is not possible for each of these proteins to bind simultaneously to the same domain on one molecule of ITSN due to steric constraints, it may nevertheless be possible for ITSNs to interact with more than one of these proteins at the same time through oligomerization of ITSNs (Figure 2). For example, a different SH3A binding protein may bind to each ITSN molecule in an oligomeric complex thereby allowing for interaction with multiple targets. However, this solution also increases the complexity of potential ITSN complexes. The related protein Eps15 forms tetramers through its coiled-coil region [14] and interacts with ITSNs through this same region ([41] and Tables S1 and S2). Since ITSN1 has been reported to have at least 24 splice variants [53] and ITSN2 has at least 4 splice variants, if we assume that each of these isoforms has the potential to form homo– or hetero-tetramers with each other and Eps15/Eps15R, then the number of potential unique tetrameric complexes that could be formed in the cell is 30^4^ or 810,000, each with potentially distinct preferences for targets! Thus, the challenge will be to define the nature and existence of such complexes and the biochemical and physiological importance of these complexes.

Our Y2H results reveal an added complexity in the biochemical pathways regulated by ITSNs. While these scaffolds play an important role in regulating endocytosis, our findings reveal a diverse array of biochemical pathways that involve these highly conserved, multi-domain scaffolds. Furthermore, these data reveal potential ITSN-regulated networks that may implicate these scaffoldings in physiological or pathological processes. Of particular interest is the potential link between ITSNs and neurological disorders. ITSN1 has been implicated in neurodegeneration (reviewed in [2]) and the identification of ITSN1 as a binding partner for DISC1 (disrupted in schizophrenia 1) suggests a potential role for ITSN1 in psychiatric disease as well [31]. Indeed, our results demonstrate that ITSN1 directly interacts with DISC1 as well as a number of proteins that either directly or indirectly interact with DISC1 (e.g., PDE4DIP, PPFIA2, RUFY, and ERC1/CAST) (Figure 5). These findings are in agreement with the recent proposal that clathrin-mediated endocytosis and protein trafficking pathways may play important roles in the pathophysiology of schizophrenia and bipolar disorder [54]. It is also interesting to note that the interactomes of DISC1 and huntingtin, the protein product of the Huntington's Disease gene, share significant overlap suggesting common modes of action for these two proteins [55]. Although ITSN1 has been directly implicated in aggregation of mutant huntingtin [56], a functional link between DISC1 and huntingtin has not been defined experimentally.

The Y2H screen performed by our lab highlights the complexity of cell signaling mediated by ITSN. Understanding ITSN binding partners provides insight into how ITSN is organizing intracellular pathways. Further exploration of the ITSN binding partners identified in this screen will aid in our understanding of cellular function.

## Supporting Information

Table S1 ITSN1 yeast two-hybrid results.
(PDF)

Table S2 ITSN2 yeast two-hybrid results.
(PDF)
