# Supplementary material for: Intersectin (ITSN) Family of Scaffolds Function as Molecular Hubs in Protein Interaction Networks
Source: PLoS One. 2012 Apr 27;7(4):e36023. doi: 10.1371/journal.pone.0036023 (PMC3338775; doi:10.1371/journal.pone.0036023)
Supplement: Table S1 — ITSN1 yeast two-hybrid results. (PDF) [file pone.0036023.s001.pdf]

Table S1. ITSN1 yeast two-hybrid results

aa coordinates	Prey Name	Accession#	Prey description	Prey-regions bound	Library screened*	Functional Pathways**
**1-118**	**EPSIN2a**	NP_683723	epsin 2a	320-422	a	1, 2
	**mEpsin2**	NP_034278	intersectin-EH binding protein 2	337-595	b	1, 2
	**CALM**	NP_009097	clathrin assembly protein lymphoid myeloid leukemia	413-652	c	1, 2
	**mPICALM**	NP_666306	phosphatidylinositol binding clathrin assembly protein	380-665	b	1, 2
	**FNBP4**	NP_056123	formin binding protein 4	468-618	a	2
	**ZFPM2; FOG2**	NP_036214	transcription factor GATA4, modulator of; friend of GATA2	417-938	a	6
	**HRB**	NP_004495	HIV-1 Rev binding protein; necleoporin-like protein	71-562	c	3, 4
	**mHRBL**	NP_835456	HIV-1 Rev-binding protein-like protein	59-479; 296-479; 342-479;305-481	b	3, 4
**1-313**	**RAB11-FIP2**	Q7L804	rab11-family interacting protein 2	98-512	a	3, 4
	**mPICALM**		phosphatidylinositol binding clathrin assembly protein	310	c	1, 2
	**Dab2**	NP_001334	disabled-2	242-770	c	1
	**HRB**	NP_004495	HIV-1 Rev binding protein; necleoporin-like protein	178-484; 512;562	c	3, 4
	**mHRB**	NP_034602	HIV-1 Rev binding protein; necleoporin-like protein	488-559	b	3, 4
	**HRB like**	NP_006067	HIV-1 Rev binding protein-like	305-481	c	3, 4
	**mPICALM**	NP_666306	phosphatidylinositol binding clathrin assembly protein	310-665	b	1, 2
**204-320**	**mEpsin2**	NP_034278	intersectin-EH binding protein 2	337-595	b	1, 2
**204-522**	**CYLN2**	NP_003379	cytoplasmic linker protein, 115 kD	716-959	a	5
	**EPS15L**	NP_067058	Eps15-like	247-489	a	1, 3
	**mEPS15L1**	NP_031970	mouse Eps15-like	169-463; 192-419	b	1, 3
	**GOLGA5**	NP_005104	golgi autoantigen, golgin subfamily a, 5	198-501	a	3, 4
	**ITSN1**	NP_003015	intersectin 1	204-334	a	1-7
	**mAMOTL1**	BAB30287	angiomotin-like 1	438-691; 430-635; 255-521; 410-548	b	5
	**mBECN1**	AAH05770	BCL2-interacting protein	34-394; 20-360; 35-251; 11-244; 171-261	b	3
	**mHMG20A**	NP_080088	High mobility group 20A	189-346	b	6

	**mKRT2-1**	NP_032499	keratin complex 2; keratin 1, type II	155-454; 194-453	b	5
	**mKRT2-6G**	NP_064340	keratin complex 2, gene 6g	185-398	b	5
	**mLOC223917**	NP_666175	hypothetical protein mLOC223917, similar to human keratin 6	161-390	b	5
	**mPPL**	NP_032935	periplakin	1125-1323; 814-1062	b	5
	**PPL**	O60437	periplakin	787-1104; 842-1258	a	5
	**mSWAP70**	NP_033328	SWAP complex protein, 70 kDa	315-516	b	3, 4
	**PIK3R1**	NP_852665	p85a phosphoinositide-3-kinase, regulatory subunit 1	309-552	a	3, 4
	**PPFIA2**	NP_003616	protein-tyrosine phosphatase, receptor type, f polypeptide-interacting protein alpha 2	359-760; 385-762	a	2-4
	**PSCD1**	Q15438	cytohesin; pleckstrin homology, Sec7 and coiled/coil domains 1	11-249	c	3, 4
	**RNF40**	O75150	Ring finger 40 Ub ligase	207-495	a	3
**350-660**	**CCDC99**	NP_060255	coiled-coil domain containing 99; Spindly protein-reg kineticores	67-288; 67-289	d	5
	**CLTC**	NP_004850	clathrin heavy chain 1	934-1177	d	1
	**DESM**	Q15787	desmin	350-469; 336-469; 127-299; 356-448; 353-429; -18-140; 297-460; 255-358; 339-448; 268-389;	d	5
	**EPS15**	NP_001972	EGFR substrate protein 15	121-390	d	1, 3
	**KIF16B**	NP_078980	kinesin family member 16B	805-957	d	1, 3, 5
**520-809**	**ARFIP2**	NP_036534	arfaptin/ POR1	29-331	c	2-4
	**DESM**	Q15787	desmin	140-465; 166-469; 254-469	d	5
	**FNBP1**	NP_055848	formin binding protein 1; FBP17	47-107	c	1-5
	**GCC1**	NP_078799	golgi coiled-coil 1	395-592	a	5
	**GOLGA5**	NP_005104	golgi autoantigen, golgin subfamily a, 5	198-501	a	3, 4
	**HIP1**	O00291	huntingtin-interacting protein 1	5-341	c	1-3
	**MTUS2/ KIAA0774**	NP_001028774	cardiac zipper protein; MT associated protein TIP150	932-1253	a	5
	**mKRT2-1**	NP_032499	keratin complex 2; keratin 1, type II	383-634; 416-615; 416-637; 214-498; 438-552; 168-537	b	5
	**mSYNC**	Q9EPM5	syncoilin	8-293	b	5
	**NY-BR-15**	NP_001035940	similar to serologically defined breast cancer antigen NY-BR-15	340-759	c	8

	**PACSIN3**	NP_057307	protein kinase C and casein kinase substrate in neurons 3	29-307; 152-298; 35-303	a	1, 2, 5
	**PDCD6IP**	NP_037506	Alix, ALG-2 interacting protein	359-540	e	1
	**PREX1**	NP_065871	P-REX-1	1012-1422	c	3, 4
	**RABEP1**	NP_004694	rabaptin-5;	466-737; 466-728	a	1, 3, 4
	**RAI14**	NP_056392	retinoic acid induced 14; novel retinal pigment epithelial gene; RAI13;	388-781	c	8
	**SCOCO**	NP_115936	short coiled-coil protein; binds Arf like protein	24-122	d	3, 4
	**TRIM8**	NP_112174	tripartite motif-containing 8; ring finger protein 27; RNF27	6-306	c	3
**709-981**	**Dab2**	NP_001334	Disabled-2	333-770	c	1
	**PI3KC2β**	Y13892	PI3K class 2β isoform	18-476	c	3
**730-816**	**Dab2**	NP_001334	Disabled-2	501-720; 242-770; 507-770; 317-722	c	1
	**PI3KC2β**	Y13892	PI3K class 2β isoform	18-475; 18-480	c	3
	**FAM59B**	NP_00116171 3.1	family with sequence similarity 59, member B,	411-609	a	8
	**mLOC244310**	NP_766498	Guanylate kinase associated protein; DLGAP2, SAPAP2	303-673	b	5
	**mPLVAP**	NP_115774	plasmalemma vesicle associated protein	123-438; 211-438	b	5
	**SPRY2**	NP_005833	sprouty 2	133-315	c	3, 4
	**mSPRY2**	NM_011897	mouse sprouty 2	31-315	b	3, 4
	**SAPAP1**	O14490	PSD-95/SAP90-associated protein 1; guanylate kinase-associated protein 1; GKAP1	4-282	a	5
	**SYNJ2b**	O15056	synaptojanin 2b	1189-1443	a	1
**807-912**	**AP2B1**	NP_001273	adaptor-related protein complex 2, beta 1 subunit, isoform b	632-937	a	1
**1064-1148**	**FCHSD2**	NP_055639	nervous wreck; NWK	465-654; 244-600; 214-584	c	2, 5
**1220-1500**	**mTIAM-1:**	U05245	T-cell lymphoma invasion and metastasis 1	470-827	b	3, 4
**1460-1500**	**mTRP51**	NP_035770	p53 tumor suppressor	26-390	b	6

*Library screened: a. brain; b. mouse embryo; c. spleen; d. skeletal muscle; e. macrophage.
**Functional Pathways: 1. Endocytosis/trafficking; 2. membrane curvature; 3. signal transduction; 4. GTPase regulation; 5. cytoskeleton; 6. nuclear function; 7. other; 8. unknown.
ITSN1 binding proteins that have not identified by previous studies are highlighted in yellow
