# Supplementary material for: Intersectin (ITSN) Family of Scaffolds Function as Molecular Hubs in Protein Interaction Networks
Source: PLoS One. 2012 Apr 27;7(4):e36023. doi: 10.1371/journal.pone.0036023 (PMC3338775; doi:10.1371/journal.pone.0036023)
Supplement: Table S2 — ITSN2 yeast two-hybrid results. (PDF) [file pone.0036023.s002.pdf]

Table S2. ITSN2 yeast two-hybrid results

aa coordinates	Prey Name	Accession#	Prey description	Prey-regions bound (aa)	Library screened*	Functional Pathways**
1-130	**AMPH**	AF099118	amphiphysin	39-273	a	1, 2
	**FNBP4***	NP_056123	formin binding protein 4	468-618	a	2
	**SNAP29**	NP_004773	soluble NSF attachment protein	6-254	c	1
	**TR4**	NP_003289	orphan nuclear hormone receptor	385-615	a	6
238-566	**AOF2**	NP_055828	amine oxidase domain 2; lysine specific histone demethylase	355-499	c	6
	**C19orf50**	NP_076974	hypothetical protein LOC79036	8-123	e	3, 4
	**CROP**	O95232	cisplatin resistance-associated overexpressed protein			6
	**EPS15***	NP_001972	EGFR substrate protein 15	4-534	c	1, 3
	**EPS15L1***	NP_067058	EGFR substrate protein 15-like protein	247-489	a	1, 3
	**ERC1**	NP_829883	Rab6 interacting protein 2/CAST	570-768	a	3-5
	**GOLGA8A**	NP_851422	golgi auto antigen 8A	61-476; 92-511; 150-381; 98-403	c	3, 4
	**GOLGB1**	NP_004478	golgi autoantiben B1	1860-2269	a	3, 4
	**HOOK2**	NP_037444		309-681	c	3-5
	**KCTD10#**	NP_114160	K-channel tetramerization domain containing 10	11-314; 8-314; 3-294	c	6
	**NBR1**	NP_005890	neighbor of BRCA1 gene 1	298-482	c	8
	**PDE4DIP**	NP_055459	phosphodiesterase 4D interacting protein, isoform 5; myomegalin	602-845	a	5
	**RNF20**	NP_062538	ub ligase	108-567;43-586	a; c	3
	**SNAP29**	NP_004773	soluble NSF attachment protein	6-254	c	1
	**STX4**	NP_004595	Syntaxin4	61-119	e	1
	**STX4A**	NP_004595	Syntaxin4	1-145	e	1
	**TACC1**	NP_006274	transforming acidic coiled-coil containing protein 1	617-711	e	5
	**TRIO**	NP_009049	Rho GEF	475-734	a	3, 4
567-805	**ANKRD17**	NP_115593	gene trap ankyrin repeat; serologically defined breast cancer antigen NY-BR-16;	203-390	a	6
	**DAPLE**	NP_001073883	DVL-binding protein DAPLE	729-912;510-911	c	5
	**DST**	NP_001714	dystonin;bullous pemphigoid antigen 1; BPAG1	1476-1616	c	5

	**ERC1**	NP_055879	Rab6 interacting protein 2/CAST	570-768	a	3-5
	**RABEP1**	NP_004694	Rabaptin-5	466-737	a	3, 4
	**ROCK1**	NP_005397	Rho kinase	627-834	c	3-5
	**RUFY1**	AAK50771	FYVE-finger protein; Rab4 interacting protein	272-459	c	3, 4
	**TMF1**	NP_009045	TATA element modulatory factor 1	567-805	c	6
**955-1180**	**AGT**	NP_000020	angiotensinogen; SERPINA8	211-485	a	7
	**AHDC1**	NP_001025053	AT hook, DNA binding motif, containing 1	49-300	c	6
	**CBL**	NP_005179	Ub ligase	485-710	c	3
	**CHIC2**	NP_036242	cystein-rich hydrophobic domain 2	44-160;64-165;	c	7
	**CPSF6**	NP_008938	ABL-associated protein 1	164-505	a	6
	**DNM2**	NP_004936	dynaminII	661-866;763-866	c	1, 3
	**FAM59B**	XP_097977	family with sequence similarity 59	411-609	c	8
	**FASLG**	NP_000630	FAS ligand	6-281	c	3, 4
	**FCHSD1**	NP_258260	Nervous wreck 2; NWK2	478-650	c	2, 5
	**FCHSD2**	NP_055639	Nervous wreck; NWK	491-665;246-425;459-609;465-652;468-676;244-600;279-656;460-601;244-600;465-654	a; c	2, 5
	**GOLGB1**	NP_004478	golgi autoantiben B1	1392-1657	a	3-5
	**GPNMB**	NP_002501	glycoprotein (transmembrane) nmb isoform b	249-336;316-385	e	8
	**HNRPK**	Q15671	heterogeneous nuclear ribonucleoprotein K, isoform b	141-440;193-463	a	6
	**ITPKA**	P23677	inositol 1,4,5-triphosphate 3-kinase A	320-461	a	3, 4
	**LARP6**	NP_060827	La ribonucleoprotein domain family, member 6, isoform 1	23-300	a	6
	**LTBP4**	O75441	latent transforming growth factor-beta binding protein 4	998-1254;953-1252;942-1255;807-1014	c	3, 4
	**MBNL1**	NP_066368	muscleblind-like; EXP	1-265	c	6
	**PDCD6IP**	NP_037506	ALIX, AIP1; ALG2 IP	682-868; 616-868	a	1
	**PIK3AP1**	NP_689522	phosphoinositide-3-kinase adaptor protein 1; BCAP:Abi interacting protein	640-804; 283-805	c; e	3
	**PIK3C2β**	NP_002637	PI3K class 2β isoform	105-552	c	3
	**RBMX**	NP_002130	RNA binding motif protein, X chromosome; hnRNP-G	44-417	a	6

	**REPS1**	NP_114128	RALBP1 associated Eps domain containing 1	434-591; 510-735; 491-736	c	1, 3, 4
	**SAPAP1**	O14490	PSD95/Dlg-assoc protein	7-288; 4-282	a	5
	**SH3KBP1**	NP_114098	CIN85	295-665	a	1, 3
	**SOS1**	Q07889	Ras GEF	1131-1207	c	3, 4
	**SOS2**	NP_008870	Ras GEF	1022-1203;738-1257	c; e	3, 4
	**SYN1**	NP_008881	synapsin I	1-146	a	1
	**SYNJ2b**	O15056	synaptojanin 2b	1100-1443	a	1
	**WASF2**	Q8IV90	Wasp family membe 4; SCAR2	21-378	c	4, 5
	**WASL**	NP_003932	N-Wasp	287-383	e	4, 5
	**WASP**	NP_000368	Wiscott-Aldrich Syndrome protein	12-396; 158-371	c	4, 5
	**WASPIP**	NP_003378	Wasp interacting peptide; WIP	266-472; 268-461; 284-477; 353-503; 275-503; 280-474	c	5
	**WIRE**	NP_573571	WASP interacting protein (WIP)-related protein,	264-440	c	5
	**YLPM1**	Q8NBB9	YLP motif containing 1	1120-1484	c	8
**1027-1110**	**FCHSD2**	NP_055639	Nervous wreck; NWK	405-579	c	2, 5
**1187-1394**	**GOLGA2**	NP_004477	golgin subfamily a, 2	245-580	c	3-5
	**PDE4DIP**	NP_055459	phosphodiesterase 4D interacting protein, isoform 5; myomegalin	727-884;651-965;749-1102; 767-967	c; e	5
	**RUFY1**	AAK50771	FYVE-finger protein; Rab4 interacting protein	421-600	c	3, 4
	**SH3GL1**	NP_003016	EEN; SH3p8	88-292	c	1, 2

*Library screened: a. brain; b. mouse embryo; c. spleen; d. skeletal muscle; e. macrophage.

**Functional Pathways: 1. Endocytosis/trafficking; 2. membrane curvature; 3. signal transduction; 4. GTPase regulation; 5. cytoskeleton; 6. nuclear function; 7. other; 8. unknown.

ITSN2 binding proteins that have not identified by previous studies are highlighted in yellow
